# Radio-Frequency Identifier Devices (RFIDs): Our Experience With Wireless Localisation in Non-palpable Breast Masses at a UK Tertiary Breast Imaging Unit

**DOI:** 10.7759/cureus.22402

**Published:** 2022-02-20

**Authors:** Chitrangada Singh, Arne Juette

**Affiliations:** 1 Radiology, Norfolk and Norwich University Hospital, Norwich, GBR; 2 Radiology, Ipswich Hospital, East Suffolk and North Essex Foundation Trust, Ipswich, GBR

**Keywords:** non-palpable breast cancer, pre-operative localization, breast cancer management, breast cancer research, breast cancer outcomes

## Abstract

The aim of this report is to evaluate the impact of the percutaneous ultrasound-guided placement of wireless radio-frequency identifier devices (RFIDs; Hologic LOCalizer, Marlborough, Massachusett) and its impact in our practice of preoperative localisation of biopsy-proven breast cancers, post-vacuum assisted biopsy-site hematoma, and lymph nodes for targeted dissection pre-operatively.

A single institutional retrospective analysis of RFID usage for preoperative localisation in screening and symptomatic patients with non-palpable biopsy-proven breast carcinoma was reviewed from the radiology information system at our tertiary breast imaging unit. Its impact on the radiological and surgical team practice was reviewed, including the number of appointments, the interval between scheduling image-guided localisation and intraoperative localisation, procedure failure, average deployment, and surgical time. Feedback from surgeons and pathologists practice was also taken into consideration.

Fifty-nine RFID clips were placed for wireless localisation of breast cancers, lymph nodes, and post-vacuum-assisted biopsy hematoma over nine months. Seventy-three per cent (73%; n=43/59) of RFID devices were placed in biopsy-proven carcinomas under direct ultrasound guidance. The learning curve was small, as the delivery system was similar to the commonly used localisation clips.

The pilot process involved RFID with radioisotope injections for the breast mass for the initial 28% (n=12 /43) cases, which were gradually transitioned into RFID only. Radioisotope was used for sentinel node purposes if required. For targeted node dissection, 3% (n=2/59) patients received RFID for biopsy-proven metastatic node localisation, with one of two with adjunct radioisotope injection. Post-vacuum-assisted biopsy (VAB) hematoma was localised in 24% (n=14/59) cases, four of which received adjunct radioisotope in the pilot phase.

The average procedure time for RFID deployment was five minutes. The average time for surgery was 20 minutes. 1.6% (n=1/59) incidence of RFID slipping from the surface of the site through surgical exposure attributed to the superficial and immediate pre-operative placement of the RFID. This was salvageable with adjunct radioisotope injection within the pilot phase. There were no incidences of repeat localisation or repeat exploration surgeries.

Planned pre-operative localisation with RFID allows for better planning and less pressured service delivery and a success rate of 98-99%. This ultimately avoids lost theatre time and patient demotivation. Surgeons have reported excellent intra-operative detectability in their approach. There has been no difficulty in the detection of the RFID within the surgical cavity despite hematoma. RFID localisers are expensive compared to our usual practice of radioisotope injection but this can be recuperated through uncoupled tariffs like gain in slots of one-stop clinics, flexibility for placement, and avoiding lost theatre time, as these can be placed up to 30 days before surgery.

## Introduction

Radio-frequency Identifier devices (RFIDs) have emerged as one of the most promising localisation techniques for not just pre-operative localisation of non-palpable breast carcinoma but also for target lymph node dissection. Interestingly, a review of the literature shows that RFID was initially introduced as a marker for localising the tip of an endotracheal tube but later found its utility in breast cancer localisation after a series of modifications.

The Breast Imaging units across the UK are getting increasingly busy with one-stop and screening assessment clinics. An additional slot of pre-operative localisation on the day of surgery with a radioisotope tracer injection or wire localisation adds extra pressure. Same-day localisation provides little flexibility in case of unexpected findings or difficult localisation [1]. Units that lack the infrastructure to support radio-tracer localisations have also been benefitted by this novel method. One of the biggest advantages of this system is its portability, which can allow for the sharing of this system in partner units.

RFIDs can be deployed as early as 30 days before the planned surgery giving the surgeons flexibility in planning their approach and surgical incision etc., thus improving the cosmesis [2]. Interestingly, the RFID retrieval rate was also 100%, which was another positive influence on the acceptability of this novel technique. This article summarises our experience with RFID placement and gradual translation from radiotracer-based localisation to RFID.

## Technical report

Standard localisation practice in our breast unit

Our breast imaging department is part of a tertiary hospital with breast screening and “one-stop” symptomatic clinics. All breast carcinomas are initially diagnosed with a 14-gauge core biopsy, followed by a surgical plan discussion at the Multidisciplinary Meeting (MDT). Cases that require localisation for lumpectomy are identified at this meeting.

Before the introduction of RFID, the usual practice for pre-operative localisation involved radioisotope injection within the biopsy-proven breast carcinoma. Isotope technetium-99m in pre-filled syringes were arranged from our nuclear medicine department on the day of surgery and injected under ultrasound guidance. This had limitations in terms that localisation was possible only on the day of surgery (within 12-24 hrs). Furthermore, masses close to the peri-areolar region often produced mixed signals from sentinel node injections.

Cases for RFID are evenly distributed in the pre-booked slots available over the week. All localisation procedures were done after informed verbal consent with particular emphasis on discomfort, bruising, hematoma formation, and possible risk of RFID displacement, which was documented in reports from post-RFID placement mammograms. The initial pilot phase included a radiotracer in adjunct to RFID to support the surgeons in comfortable transition. A hydro-mark clip was primarily placed in mammographically occult carcinomas and ductal carcinoma in situ (DCIS) at the time of initial biopsy before RFID placement and was used as a surrogate.

All RFID placements were done under ultrasound guidance by consultant breast radiologists, consultant radiographers, and breast radiologists (fellows) using the GE LOGIC P9 ultrasound machine (General Electricals, Boston, Massachusetts) under local anaesthesia. Post-procedure mammograms (including craniocaudal and oblique views) were obtained to demonstrate the relationship of the RFID from the targeted abnormality. The minimum depth of the RFID from the skin surface was detected using the wireless handheld “Readout” device and mentioned on the reports.

Surgeons had access to all the procedure-related imaging as well as the reports. An intra-operative sterile single-use probe with an 8 mm fine tip assisted in intra-cavitary localisation connected to the handheld device assisting the surgeons in intra-operative depth assessment and planning. Specimen radiography was used to confirm and document the presence of RFID. A formal pathological analysis of the specimen was used to confirm the RFID site and localised abnormality within the lumpectomy specimen.

Materials and methods

This was a single-institution retrospective study, including 59 cases with RFID usage for localisation between December 2019 and August 2020. This included screening-detected as well as symptomatic patients with non-palpable biopsy-proven carcinoma. The data were collected from the radiology information system (RIS). The cases were anonymised for patient confidentiality. The data were analysed in terms of findings and case-specific utility. The impact of this new localisation method in surgical and pathological specimen handling was also analysed in form of feedback and surgical outcome.

Device Specifications

**Figure 1:**
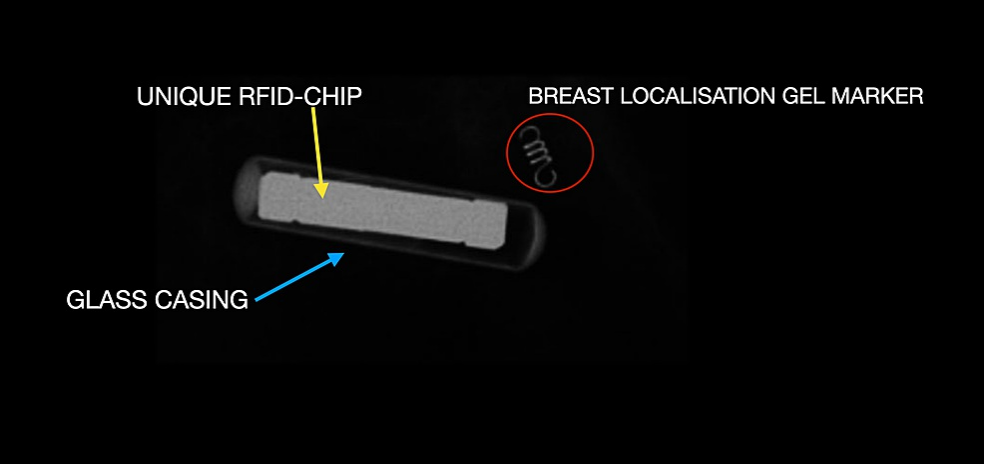
Structure of an RFID The magnified radiograph of an RFID device and HydroMark® clip (Mammotone, Cincinnati, Ohio). The chip (yellow arrow) can be seen encased in a glass casing (blue arrow). The total unit measures 9 mm. The commonly used Gel Mark clip can be seen for comparison of size and appearance (red circle). RFID: radio-frequency identifier device

The Hologic RFID TAG device system (Hologic, Marlborough, Massachusett) constitutes a single-use 12-gauge needle delivery system with a pre-loaded RFID localisation clip and 10 cm length. Each preloaded RFID clip has a unique number chip encased within a glass shell and measures up to 9 mm. (Figure 1). For surgical usage, a battery-operated reusable handheld wireless device that can detect signals up to 60 mm is employed. An intra-operative sterile single-use probe with an 8 mm fine tip is also provided for intra-operative detection.

Patient Specifications

**Figure 2:**
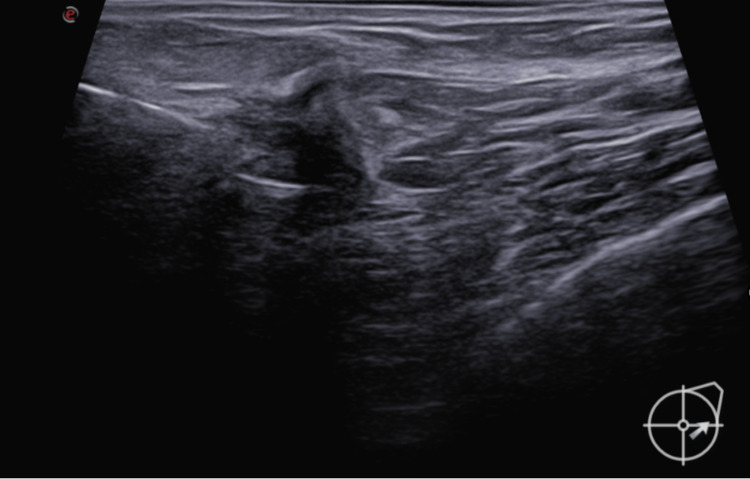
RFID usage Under local anaesthesia, the introducer needle with the notch facing upwards ~(like a step defect) is introduced within the tumour under ultrasound guidance. RFID: radio-frequency identifier device

**Figure 3:**
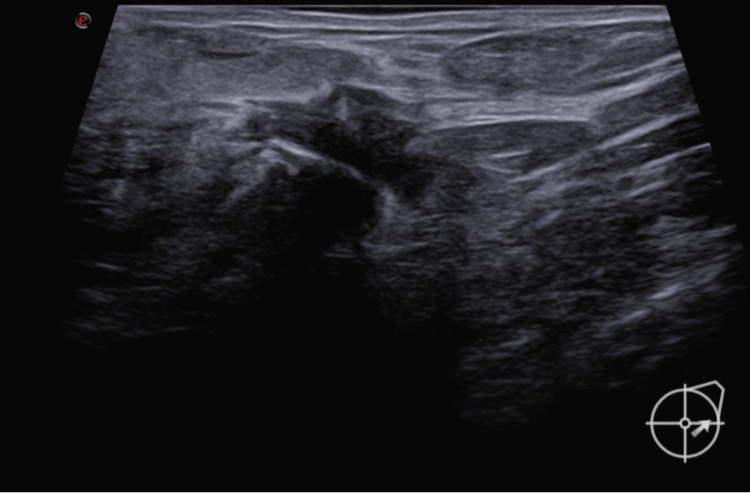
RFID usage: post-deployment Once the needle is in a good position, the RFID is deployed within the tumour under ultrasound guidance, as seen in the figure. RFID: radio-frequency identifier device

Inclusion criteria: Women with biopsy-proven, non-palpable breast carcinoma or DCIS suitable for wide local excision were included for this method of localisation. Targeted lymph node dissection cases were also included for biopsy-proven metastatic nodes. The localised abnormality was suitable to identify on mammography and ultrasound. All RFID placement was done under ultrasound guidance only during this study. Five ml of 2% lidocaine was used as a local anaesthetic (Figures 2-3).

Exclusion criteria: Patients that required possible further follow-ups or localisation using breast MRI were excluded as RFIDs are MR conditional and known to cause susceptibility artefacts up to 80 mm.

Results

**Figure 4:**
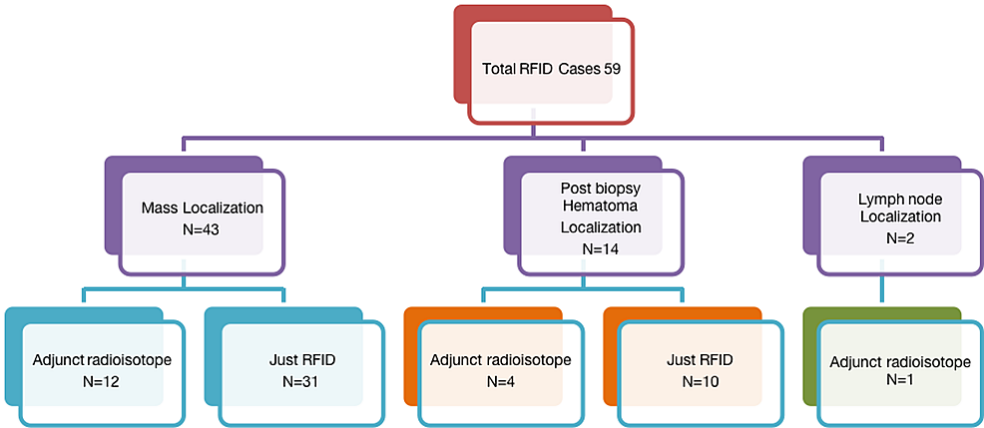
Datasheet Statistics from our single institutional retrospective study

**Figure 5:**
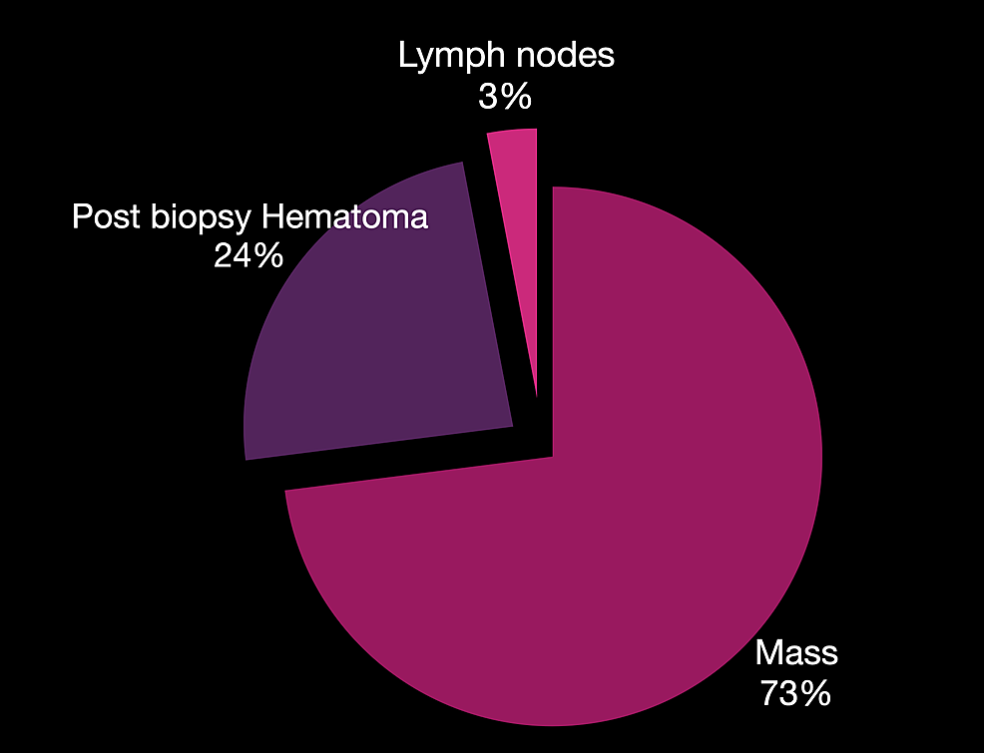
Placement sites for RFID included in our study. Mass: RFID placed into or adjacent to the ultrasound mass abnormality. Hematoma: RFID placed within a post-stereotactic biopsy hematoma. Lymph node: RFID used for localising the target lymph node. RFID: radio-frequency identifier device

A total of 59 RFID clips were placed for wireless localisation of breast cancers, lymph nodes, and post vacuum-assisted biopsy hematoma over nine months. All the RFID devices were placed under direct ultrasound guidance. The learning curve was small, as the delivery system was similar to the commonly used localisation clips. Initial experiences reflected the adaptation of good skin incision as the introducer needles were a 12-gauge bevelled end (wider than the usual 14-gauge clip delivery needles with sharp edges). The pilot phase involved using a combination of radioisotope with RFID in 29% of cases (n=17/59) (Figures 4-5).

A total of 73% (n=43/59) of RFID devices were placed in biopsy-proven carcinomas under direct ultrasound guidance. The pilot process involved RFID with radioisotope injections for the breast mass for the initial 28% (n=12/43) cases, which were gradually transitioned into RFID only. Radioisotope was used for sentinel node purposes if required. All the localisations were for biopsy-proven carcinoma. No benign mass was localised during our study. For targeted node dissection, 3% (n=2/59) patients received RFID for biopsy-proven metastatic node localisation, with one of two with adjunct radioisotope injection. A post-VAB hematoma was localised in 24% (n=14/59) cases, four of which received adjunct radioisotope in the pilot phase. The average procedure time for RFID deployment was five minutes.

The average time for surgery was 20 minutes. There has been no reported complaint about any discrepancy in the estimation of distance from the probe to RFID in our study. There was a 1.6% (n=1/59) incidence of RFID slipping from the surface of the site through surgical exposure attributed to the superficial and immediate pre-operative placement of the RFID. This was salvageable with adjunct radioisotope injection within the pilot phase. There were no incidences of repeat localisation or repeat exploration surgeries. The retrieval rate was 100%. Both surgeons and radiologists have preferred RFID over wire localisation. There was also satisfactory patient feedback.

## Discussion

Non-palpable breast carcinoma constitutes not just a majority of screen-detected cases but also occult carcinomas and has always required a reliable pre-operative percutaneous localisation method for assisting breast surgeons. Despite the presence of wire-based localisation systems that often accompany possible displacement and intra-operative transection issues [3], technetium-based radioisotope tracer injections demand facilities for production, injection, and processing of the sample; there has been a constant need for innovation. Even the magnetic seeds have gained popularity but RFIDs have emerged as winners with the ability to localise more than one site and the ability to distinctly identify the RFID by their number and depth from the surface, guiding the surgeon’s even intra-operatively. 

RFIDs have been used for localising breast carcinomas since 2015 but still carry a scope for the localisation of lymph nodes for targeted excision. The first single largest trial was conducted by a European study by Malter et al. including 147 patients [2]. 

A study conducted by Wazir et al. in Princess Grace Hospital, London, in 2019 included a small cohort of 10 patients with the deployment of 11 RFID tag localisers under ultrasound guidance [1-2]. The outcome included seven malignant and four benign masses, unlike our study where lymph nodes were also localised in 13% (N = 2/59) cases for targeted excision. After its initial success and supportive evidence collected for this paper, it has been adapted and used more readily [4]. Most recently, in December 2020, Lowe’s et al. published a study including lymph node localisation using RFIDs [5]. Further, with our unit’s experience, we noticed better outcomes with the placement of the RFIDs at the deeper aspect of the target instead of the subcutaneous site, which may dislodge during surgical incisions and exposures. Their mean time was comparable to our study, with an average range between five and 10 minutes and a mean surgical time of 20 minutes.

All RFID devices in our study had audible signals during the placement as well as intra-operative localisation. All lumpectomy specimens demonstrated clear margins on pathology with no instance of re-localisation.

**Figure 6:**
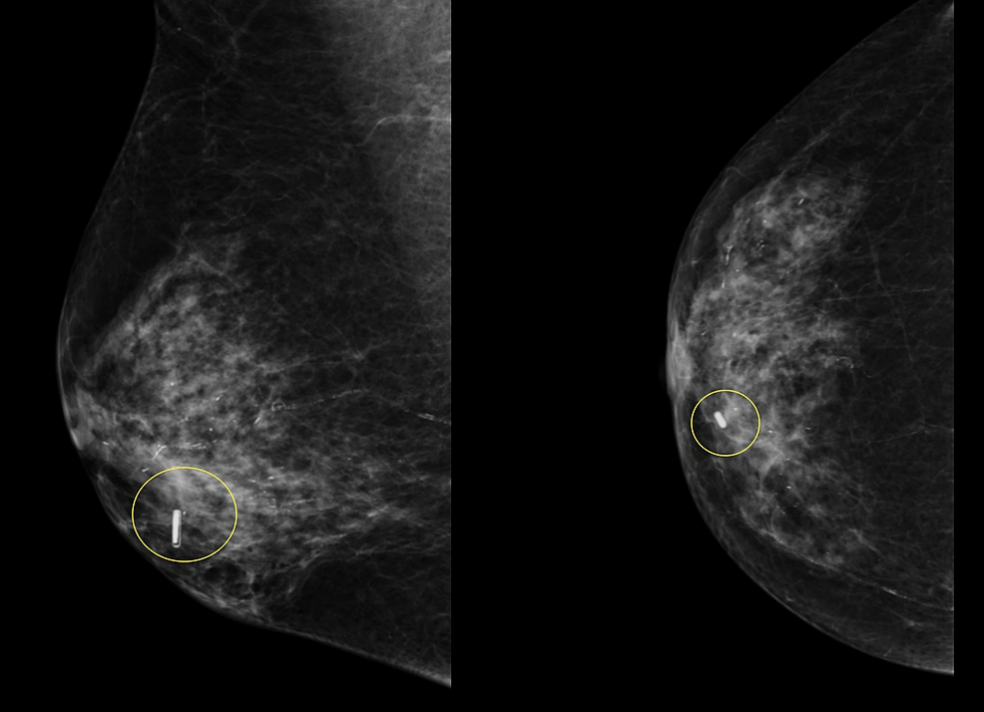
Wireless localisation of periareolar masses does not hinder sentinel node radio-isotope injections This case demonstrates the excellent utility of RFID in periareolar/retro areolar masses, which also require sentinel node injections. Localising both areas with isotopes can give mixed signals and may lead to failed localisation. RFID: radio-frequency identifier device

In a nutshell, the window of localisation and surgery provided us with better planning and spaced out appointments. The loss of theatre time due to same-day localisation was also recovered and rescheduling of surgeries was easily possible. With feedback from surgeons, they were very impressed by the ability to localise retro-areolar masses using RFID LOCalizers and simultaneously differentiate between the peri-areolar radiotracer injection signal for sentinel node localisation (Figure 6).

**Figure 7:**
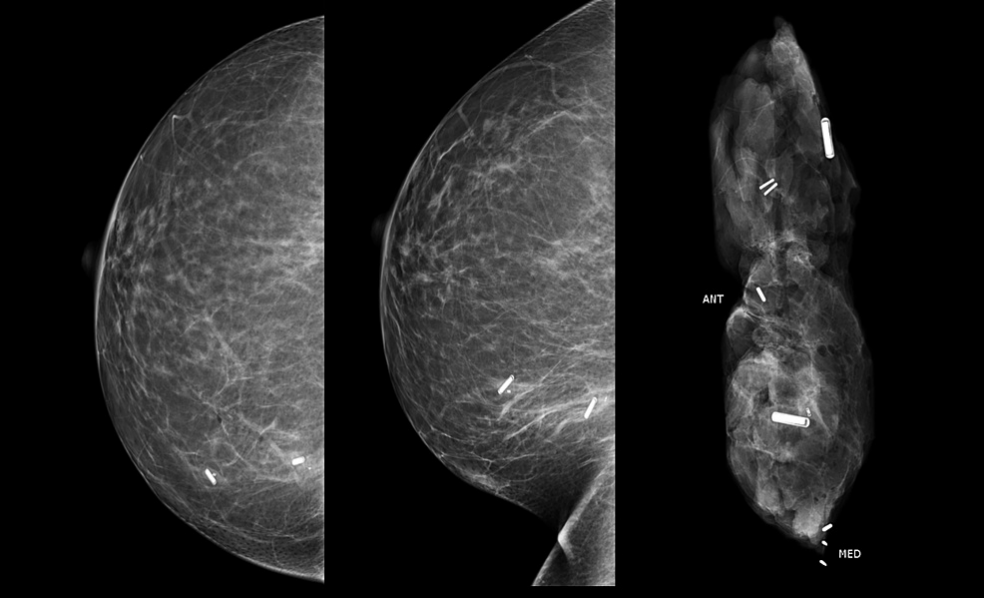
Benefit of a unique RFID chip in each localiser This case demonstrates the excellent utility of RFID in multiple localisations even within the same quadrant of the breast. RFID: radio-frequency identifier device

Multiple RFIDs, up to two, were also included for the localisation of double lumpectomies in our study. Each RFID could be distinctly identified intra-operatively using the RFID number and on the specimen radiograph (Figure 7).

**Figure 8:**
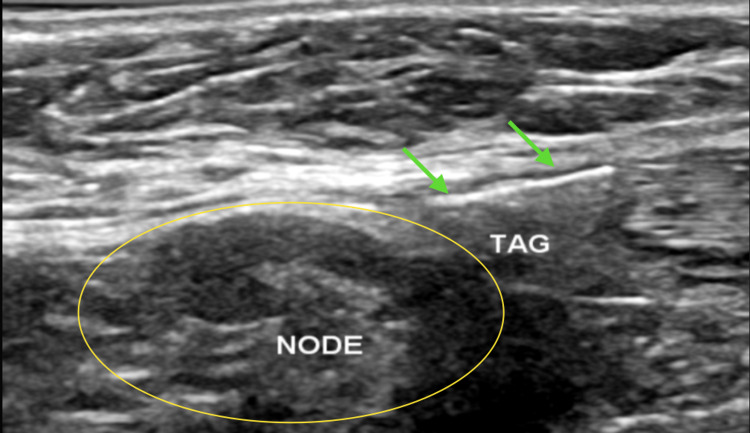
Beyond the scope of tumour localisation We started with at least two successful metastatic lymph node localisations for targeted lymph node dissection purposes in the pilot phase. The figure shows the RFID tag (green arrows) deployed just adjacent to the metastatic lymph node (yellow circle). RFID: radio-frequency identifier device

In one of the largest trials at Massachusetts General Hospital (USA), published in 2021, for radio frequency-aided localisation, using at least 1013 RFID over 848 patients, only breast lesions were targeted, but in our study, target lymph nodes localisation was also included, which was an additional benefit [6]. The RFID placement was decided based on the size of the axillary lymph node. In very small but solitary and biopsy-proven metastatic nodes, RFID was placed adjacent to or just under the node. With enlarged, completely replaced nodes, RFID could be deployed into the node itself (Figure 8). This has been helpful in following up these nodes with a post-chemotherapy response and ensured confirmed excision on surgery.

Limitations of RFID localisation devices

The current guidelines and licensing advocate RFID tag deployment within 30 days of surgery by the European Union but the USA permits long-term deployment. Large-scale studies are still awaited. Phantom artefacts of up to 20 mm limit MRI monitoring for neoadjuvant chemotherapy (NACT) patients. Contrast-enhanced spectral mammography (CESM) can be a proposed alternative for such patients, but we have limited experience and data so far. 

## Conclusions

To summarise, RFID systems have established popularity and confidence, not only amongst breast radiologists but have also become a widely accepted method for pre-operative localisation by surgery and pathology teams. With a 100% retrieval rate, as in our study, it has replaced the conventional wire and radio-isotope localisation in the majority of units, including tertiary as well as district general hospitals across the UK.

The introduction of RFIDS has furnished the breast units with flexibility in pre-operative localisations as a scheduled appointment (up to 30 days prior), rather than a 'same-day' procedure. This method has also benefitted smaller departments that lacked the infrastructure for radiotracer manufacturing and handling facilities with this portable, wireless device. Patients are also comfortable with early localisation, as they don't have to rush to the imaging/nuclear medicine department just before their surgery for wires or radiotracer injection. This relieves last-minute anxiety and procedural delays due to possible localisation/equipment failure.
